# Psychometric Properties of the Korean Version of the Smoking Media Literacy Scale for Adolescents

**DOI:** 10.3389/fpubh.2021.675662

**Published:** 2021-06-23

**Authors:** Sookyung Kim, Hyeonkyeong Lee, Jung Jae Lee, Hye Chong Hong, Seungjoo Lim, Junghee Kim

**Affiliations:** ^1^College of Nursing, Yonsei University, Seoul, South Korea; ^2^Mo-Im Kim Nursing Research Institute, College of Nursing, Yonsei University, Seoul, South Korea; ^3^Li Ka Shing (LKS) Faculty of Medicine, School of Nursing, The University of Hong Kong, Hong Kong, China; ^4^Department of Nursing, Chung-Ang University, Seoul, South Korea; ^5^Department of Nursing, Research Institute for Basic Science, Hoseo University, Asan, South Korea; ^6^Department of Nursing, Yonsei University Wonju College of Medicine, Wonju, South Korea

**Keywords:** smoking, media literacy, adolescent, validation, adaptation

## Abstract

Smoking media literacy has proven to be an effective competency for reducing adolescents' smoking. This study aimed to cross-culturally modify the smoking media literacy scale and evaluate the validity and reliability of the Korean version of the revised Smoking Media Literacy Scale for Adolescents (K-SMLS). The translation of the K-SMLS was conducted according to the World Health Organization's guidelines. After the translation process, an online survey was conducted with convenience samples of 215 total adolescents from five high schools in the capital city of Korea. Construct validity was examined by exploratory factor analysis and confirmatory factor analysis. Internal consistency reliability was examined with Cronbach's alpha. The final version of the K-SMLS consisted of 15 items. The goodness of fit, determined through a confirmatory factor analysis of the three domains, was acceptable [χ^2^ = 237.85 (*p* < 0.001), CFI = 0.93, TLI = 0.92, RMSEA = 0.09, SRMR = 0.09]. The reliability of the K-SMLS was satisfactory (Cronbach's alpha = 0.78). The findings provide evidence for a valid and reliable tool that can be used to assess smoking media literacy in Korean adolescents. Further studies with a probability sampling design are suggested as the use of convenience samples limits the generalizability of the results to other populations.

## Introduction

Adolescent smoking is a global health concern (1). Although the prevalence of tobacco use has declined in most countries with a high human development index (2), the continued diversification of tobacco products, such as electronic cigarettes (hereinafter e-cigs), and tobacco industry's novel marketing strategies through social media have significantly contributed to adolescents' imitation of tobacco smoking (3, 4). In South Korea (hereinafter Korea), adolescent smoking is on the decline, as cigarette use was reported to be only 6.7% in 2019 (5), which is significantly lower than in other developed countries (OCED average: 11.7%) (6). However, there is a growing interest in emerging tobacco products, such as e-cigs and heat-not-burn products, while the influence of smoking-related media has increased in recent years (7–9).

The considerable impact of the media on adolescents has been widely reported. Researchers have demonstrated that adolescents develop permissive attitudes toward dangerous behaviors after seeing depictions of smoking in the media (10). Similarly, adolescents develop positive attitudes toward smoking after seeing their peers post smoking-related content on social media (11). Since depictions of smoking in the media induce curiosity about smoking among adolescents, the tobacco industry has aimed for their products to gain exposure in the media including on television, in movies, and on social media, often by targeting young people with attractive images (12). Recently, social media has become an important marketing platform for the tobacco industry (13, 14), while the sharing and viewing of user-generated content (i.e., *selfies*) that depict smoking have contributed to its normalization (15). In Korea, smoking scenes appear in more than 50% of web-based cartoons, movies, and dramas that Korean adolescents enjoy (16). Further, after an analysis of popular YouTube channels, researchers found that 72.7% of videos displayed tobacco products or smoking, while 86% of channels showed prominent *YouTubers* smoking (16).

Smoking media literacy (SML), defined as the understanding, analysis, appraisal, and interpretation of media messages about smoking (17), has been accepted as an important concept in addressing adolescent smoking. According to a systematic review by Vahedi et al. (18), interventions for enhancing media literacy were found to be effective in mitigating risky health behaviors, such as smoking, among adolescents. Additionally, a large cluster-randomized trial showed that high school students (14–15 years old) who received SML education for 5 weeks had a significantly higher tendency to perceive a reduction of the smoking rate compared to those without SML education (19). In Korea, a partial amendment to the National Health Promotion Act, which regulates the promotion of cigarettes, tobacco-like products, and e-cigs, was established at the State Council in 2020 (20). Although regulations for adolescent smoking are being reinforced, reducing adolescents' exposure to depictions of smoking in the media has been a difficult process because of the emergence of new tobacco products and tobacco companies' changing marketing strategies (21, 22). E-cigs are now marketed indirectly by social media influencers rather than via traditional marketing means (21). Therefore, SML, which has proven to be an effective strategy to reduce adolescent smoking, should be the focus of future efforts to prevent smoking and lower smoking rates.

To assess SML and general media literacy among adolescents in the United States, the Smoking Media Literacy Scale for Adolescents (SMLS) was developed by Primack et al. (17) and was later revised in 2014 (19). The scale has been used in Hungary (23) and Vietnam (24). However, due to recent changes in information and communication technologies, it is necessary to modify the scale to reflect the current state of social media. This study aimed to (i) cross-culturally modify the SMLS and (ii) evaluate the validity and reliability of the Korean version of the Smoking Media Literacy Scale for Adolescents (K-SMLS).

## Materials and Methods

### Translation of SMLS

#### SMLS

The SMLS is a one-factor scale with 18 items. The scale's items are classified into three core domains: (i) Authors and audiences (items 1, 2, 3, and 4); (ii) Messages and meanings (items 5, 6, 7, 8, 9, 10, 11, 12, and 13); and (iii) Representation and reality (items 14, 15, 16, 17, and 18). The 18 items are scored on a 4-point Likert-type scale (0 = *strongly disagree*, 1 = *disagree*, 2 = *agree*, and 3 = *strongly agree*). Total raw scores range from 0 to 54. The total scores were converted to a 10-point scale by dividing the raw score for the 54-point scale by 5.4. Following the SMLS' authors, we also converted K-SMLS scores to a 10-point scale by dividing the raw score for the 45-point scale by 4.5. The SMLS exhibited a Cronbach's alpha of 0.87. This study was conducted after obtaining approval from the original scale's corresponding author to develop the K-SMLS.

#### Translation and Adaptation

**Figure 1:**
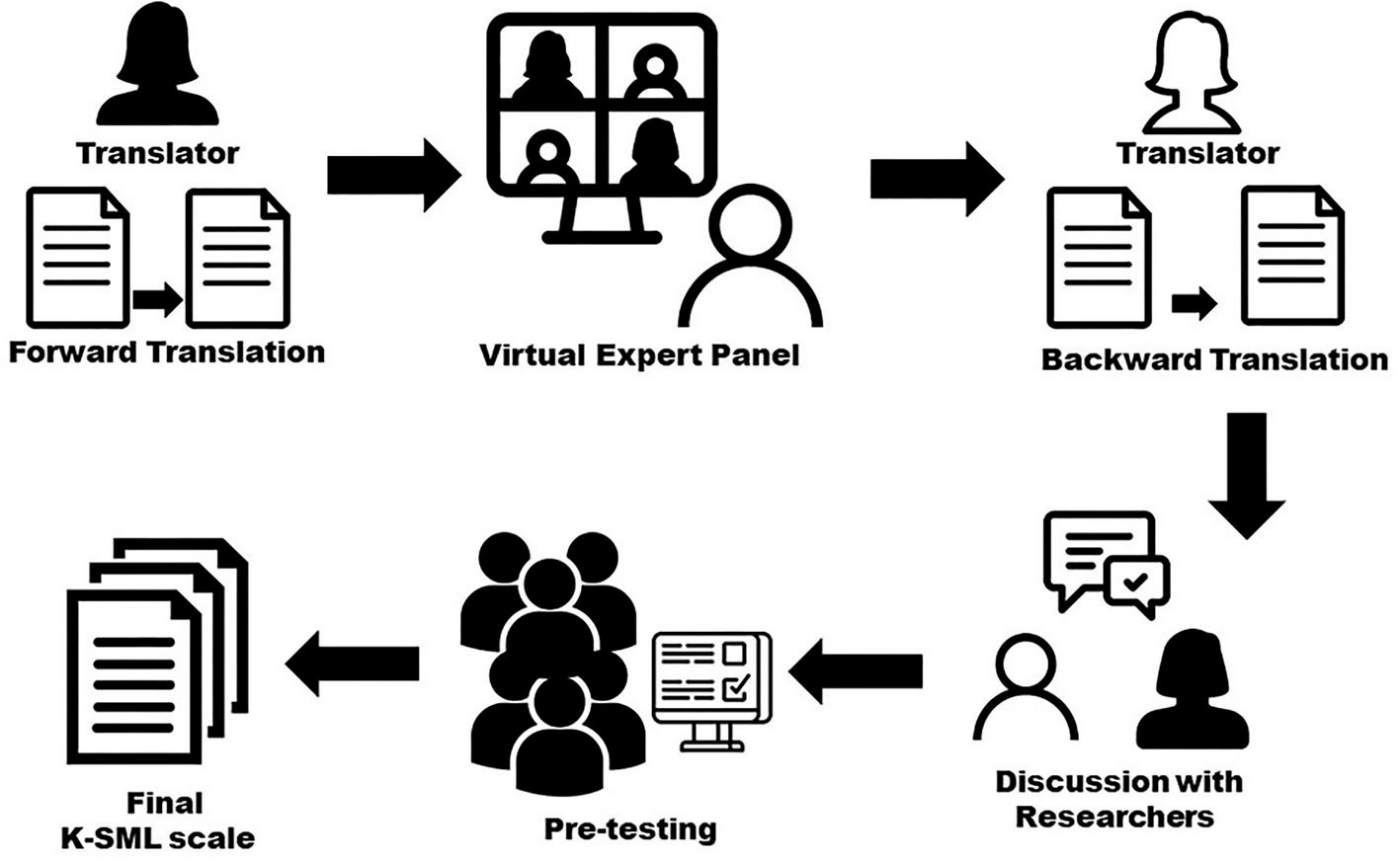
Translation and adaptation process.

The translation and adaptation process of the SMLS was performed in five steps (25) (Figure 1). In the initial step, a bilingual translator, fluent in both Korean and English, translated the English version of the SMLS into Korean. In the second step, the translated scale was reviewed and revised for accuracy and cultural relevance with help from a virtual panel of five experts (including one translator, two health care professors, the corresponding author, and the first author of this study). According to Article 10 of the enforcement decree of the Tobacco Business Act in Korea, the acts of providing bounty for tobacco sales, premiums, merchandise coupons, and other money or goods are prohibited (26). Thus, considering the current regulations on the promotion of tobacco sales in South Korea, three items were deleted (items 1, 5, and 16) based on an expert meeting with four professors who had experience in instrument development and translation. The removed items were as follows: Item 1—“Buy-one-get-one-free” deals on cigarettes are designed to get people addicted; Item 5—Wearing a shirt with a cigarette logo on it makes you into a walking advertisement.; Item 16—When you see a “buy-one-get-one-free” cigarette deal, it's usually not actually a good deal in the long run. The term “ads” was changed to “social media (*YouTube, Instagram*, etc.) promotion,” as social media posts promoting smoking are not regulated yet in South Korea. Subsequently, another bilingual translator who had not seen the SMLS translated the Korean version back into English (i.e., back-translation). Finally, the corresponding and first author of this study reviewed the original scale and the back-translation, discussed sections that appeared unclear, and corrected the translation. The final version of the K-SMLS was pre-tested with six adolescents aged 15–18 years old *via* an online survey to ensure that all items were understandable, appropriate, and culturally sensitive. Most participants responded without any difficulty. They understood that the term “social media (*YouTube, Instagram*, etc.) cigarette promotion” included videos, images, and posts reviewing tobacco products (e.g., cigarettes and e-cigs).

### Content Validation

We used the item-level content validity index (I-CVI) (27) to evaluate whether the K-SMLS reflected the meaning of the SMLS' items appropriately, which allowed us to validate our version of the scale. A panel of nine experts was formed, comprising three professors in the health care field, three high school health teachers, and three master's or PhD candidates studying adolescent smoking. These experts were asked to rate the relevance of the scale's 15 items on a 4-point Likert scale (where 1 = *not relevant*, 2 = *somewhat relevant*, 3 = *quite relevant*, and 4 = *very relevant*). Each item's I-CVI was calculated as the proportion of experts who answered that the item was either *quite relevant* or *very relevant*. If the I-CVI value of an item was >0.8 (27), the item was deemed valid. Afterward, the experts were asked open-ended questions on the items' comprehensiveness.

### Psychometric Properties

A psychometric evaluation of the K-SMLS was conducted to assess its construct validity and its internal consistency.

#### Sample and Data Collection

For this study, participants were conveniently sampled from five high schools in the capital city of South Korea. Potential participants were provided with information regarding the study's purpose; they were informed that their participation was voluntary, as they had the right to withdraw from the study at any time. It was also explained that their decisions whether or not to participate would not affect any school activities. Subsequently, informed consent forms were distributed to potential participants and their parents by teachers who were trained data collection process. Students who voluntarily wanted to participate brought research information and informed consents to their parents at home, and both adolescents and parents signed informed consents and submitted them to the teacher. The informed consents included a cell phone number to receive an online survey link. A total of 298 adolescents who submitted informed consents were invited to an online survey by giving the survey link. Two hundred fifty-four participants completed the online survey between August 4 and 21, 2020. A total of 39 surveys were excluded because they were outliers (*n* = 5) or duplicate submissions (*n* = 34). Ultimately, 215 valid surveys were identified, and response rate was 72.1%. The anticipated sample size was over 150 based on the criteria for the factor analysis, with a ratio of at least 5–10 cases per item (28).

#### Statistical Analysis

Data were analyzed using SPSS software for Windows (version 25, IBM Corp., Armonk, NY, USA) and RStudio (version 4.0.3., R Core Team, Vienna, Austria). A descriptive statistical analysis was conducted to parse participants' demographic characteristics, SML, and susceptibility to smoking. To assess the construct validity, an exploratory factor analysis (EFA) and a confirmatory factor analysis (CFA) were performed. We used a parallel analysis (PA), the eigenvalues-greater-than-one rule, and a screen test for factor extraction at first. PA requires carrying out a comparison of the eigenvalues from actual study data with randomly generated eigenvalues. The number of factors to retain is equal to the number of actual study eigenvalues that exceed randomly produced eigenvalues. We carried out a PA with 1,000 random datasets and the 95th percentile of eigenvalues in this study. EFA was conducted to support construct validity by identifying the characteristics of the factors. Subsequently, CFA was performed to assess the model's fit. CFA included root mean square error of approximation (RMSEA), comparative fit index (CFI), Tucker-Lewis Index (TLI), and a chi-squared test. The RMSEA is a measure of average residual variance and covariance; average models have RMSEA values of ≤0.09. The criteria for accepted RMSEA value ranges are as follows: 0.05–0.08 (fair), 0.08–0.10 (mediocre), and >0.10 (poor) (29). The CFI is an index ranging from 0 to 1, with a value of >0.90 considered to be an indicator of good model fit (29, 30). To identify the internal consistency of the Korean version, we assessed Cronbach's alpha for total items as a whole and confirmed the item-total correlation coefficients.

### Ethical Considerations

The study was approved by the Institutional Review Board of Yonsei University prior to conducting the study (IRB No. Y-2020-0066). All participants were provided with information about the study, signed an informed consent form, and received remuneration for participating in the study.

## Results

### Demographic Characteristics of Participants

Table 1 summarizes participants' demographic characteristics. Participants' mean age was 16.73 ± 0.79 years. Male participants comprised 64.7% of the sample, while 14% of the participants reported having smoking experience. Further, 3.3% of the participants were current smokers, while 25.6% were classified as susceptible to smoking. About 71.2% of the participants' parents were current or past smokers, while 53.5% of the participants' friends were current or past smokers. Moreover, 16.3% of the participants perceived their friends to be heavy smokers, while 37.2% perceived them to be moderate smokers. Additionally, 30.2% of the participants spent over 4 h using their smartphone and/or computer daily. More than 70% of the participants' fathers and 67% of their mothers had higher education.

**Table 1:** Demographic characteristics of the participants (*n* = 215).

**Variables**	**Categories**	**Total**
		**Mean ± SD or *n* (%)**
Age (year)		16.73 ± 0.79
Gender	Male	139 (64.7)
	Female	76 (35.3)
Smoking experience	Yes	30 (14.0)
	No	185 (86.0)
Current smoker	Yes	7 (3.3)
	No	208 (96.7)
Susceptibility to smoking	Yes	55 (25.6)
	No	160 (74.4)
Parents' smoking	Yes	153 (71.2)
	No	62 (28.8)
Friends' smoking	Yes	115 (53.5)
	No	100 (46.5)
Perceived level of best friends' smoking	Heavy smoking	35 (16.3)
	Moderate smoking	80 (37.2)
	No smoking	100 (46.5)
Daily usage of smartphone and computer (hours)	1–3	102 (47.4)
	3–4	48 (22.3)
	Over 4	65 (30.3)
Father's education	Middle school and below	3 (1.4)
	High school	37 (17.2)
	University and above	155 (72.1)
	Do not know	20 (9.3)
Mother's education	Middle school and below	2 (0.9)
	High school	40 (18.6)
	University and above	144 (67.0)
	Do not know	29 (13.5)

### Content Validity

The 14 items exhibited an I-CVI value of ≥0.78 among 15 items. Item 4, which asserted that cigarette ads link smoking to things that people want (such as love, good looks, and power) exhibited an I-CVI value of 0.56. However, we judged it appropriate to include the respective item in the K-SMLS because it fit the SMLS from a conceptual standpoint.

### Construct Validity

According to the EFA of the 15 items, the result of the Kaiser-Meyer-Olkin test was 0.79, and Bartlett's chi-squared test of sphericity with statistical significance was <0.001, which indicates that the factor analysis of these data was appropriate. The EFA was performed using a generalized least-squares technique due to a ceiling effect. Oblique rotation was conducted by considering the characteristics of K-SMLS that tend to be correlated with each item. Although the original scale had a one-factor model, the three sub-concepts of the scale showed the validity of results. In this study, the PA revealed that the eigenvalues of the three factors were bigger than the 95th percentile in the distribution of eigenvalues derived from the random data. Although 10 of the 15 items had factor loadings >0.30, five items were retained as they were conceptually linked to the scale (Table 2).

**Table 2:** Results of the exploratory and confirmatory factor analyses (*n* = 215).

**Domain**	**Item**	**EFA**			**CFA**		
		**Factor 1**	**Factor 2**	**Factor 3**	**Factor loading**	**AVE**	**CR**
Authors and audiences	1. Tobacco companies are very powerful, even outside of the cigarette business (e.g., ginseng, sports club management) (2).	0.223			0.413	0.26	0.51
	2. Tobacco companies only care about making money (3).	0.257			0.449		
	3. Certain cigarette brands are designed to appeal to younger people (4).	0.376			0.649		
Messages and meanings	4. Social media (*YouTube, Instagram*, etc.) cigarette promotion link smoking to natural things that humans want like love, good looks, and power (6).		0.255		0.414	0.40	0.83
	5. Two people may see the same movie or TV and get very different ideas about it (7).		0.546		0.726		
	6. Two people may see the same social media (*YouTube, Instagram*, etc.) cigarette promotion and get very different ideas about it (8).		0.782		0.691		
	7. Cigarette signs/advertisements in convenience stores may catch one person's attention but not even be noticed by another person (9).		0.283		0.446		
	8. People are influenced by TV or movies, whether they realize it or not (10).		0.978		0.865		
	9. People are influenced by social media (*YouTube, Instagram*, etc.) cigarette promotion whether they realize it or not (11).		0.508		0.802		
	10. When people make TV or movie, every camera shot is very carefully planned (12).		0.285		0.331		
	11. There are often hidden messages in social media (*YouTube, Instagram*, etc.) cigarette promotions (13).		0.507		0.576		
	12. Most movies or TV that show people smoking make it look more attractive than it really is (14).		0.362		0.565	0.36	0.69
Representation and reality	13. Social media (*YouTube, Instagram*, etc.) cigarette promotion show green, natural, healthy scenes to make people forget about the health risks (15).			0.378	0.549		
	14. When you see a social media (*YouTube, Instagram*, etc.) cigarette promotion, it is very important to think about what was left out of the promotion (17).			0.508	0.640		
	15. Social media (*YouTube, Instagram*, etc.) promotion usually leave out a lot of important information (18).			0.346	0.659		
	Eigenvalue	4.07	1.51	1.44			
	Explained variance (%)	27.1	10.0	9.60			
	Cumulative (%)	27.1	38.2	46.8			
	Kaiser-Meyer-Olkin (KMO) = 0.79 Bartlett's test of sphericity = 725.32 (*p* < 0.001)				Model fitness χ^2^ (87) = 237.85, *p* < 0.001, RMSEA = 0.09, SRMR = 0.09, CFI = 0.93, TLI = 0.92		
	Total Cronbach's α = 0.78						

*The parentheses indicate the item number of the original scale. EFA, Exploratory factor analysis; CFA, Confirmatory factor analysis; AVE, Average variance extracted; CR, Composite reliability; RMSEA, Root mean square error of approximation; SRMR, Standardized root mean square residual; CFI, Comparative fit index; TLI, Tucker-Lewis index*.

The R-lavaan package was used to perform CFA by incorporating Diagonally Weighted Least Squares (DWLS) as an estimator to examine our model's fit because the K-SMLS has ordinal variables and our data were positively skewed. The findings showed that the model's fit was good: χ^2^ = 237.85 (*p* < 0.001), CFI = 0.93, TLI = 0.92, RMSEA = 0.09, SRMR = 0.09 (Table 2). The average variance extracted (AVE) and composite reliability (CR) were calculated for every domain; however, one domain did not meet the minimum cutoff of the CR of 0.6 should the AVE be less than 0.5 (31). The value of CR was slightly lower than expected, but it was still acceptable because the model was fit.

### Reliability

The Cronbach's alpha of the SMLS was 0.87 (17). In our study, the Cronbach's alpha of the K-SMLS was 0.78. Additionally, the reliability was 0.78 as per McDonald's Omega values. The item-total correlation coefficients were > 0.30 except for item 10 (ranging from 0.303 to 0.537) (Table 2).

## Discussion

As the impact of media literacy on the health behavior of adolescents is increasing, a scale to measure SML that reflects the current situation in smoking behavior research is needed. This study described the cross-cultural translation process of the revised SMLS into Korean and examined the K-SMLS' psychometric properties in accordance with Korea's tobacco policy and environment of increased tobacco promotion on social media. The findings indicate that the K-SMLS is a valid and reliable instrument to assess SML among Korean adolescents. To maintain cross-cultural and conceptual accuracy, we performed a rigorous translation process that included contributions from an expert panel, back-translation, and a trial with high school students.

The findings confirmed that the K-SMLS has acceptable internal consistency (Cronbach's alpha = 0.78). The item-total correlation coefficients were higher than 0.30, indicating that the revised version is acceptable and item discrimination is appropriate (30).

We tested the construct's validity using EFA and CFA and found that the K-SMLS could adequately measure adolescents' SML. Although several items showed low factor loading, there was no need to exclude any items because our study's goal was to confirm the translated version of the SMLS, not to reduce the number of items to develop a new scale. During CFA, the RMSEA achieved desirable values, while the CFI and TLI values were also satisfactory.

Although a factor loading of 0.32 is acceptable in socio-behavioral studies (32), the relatively low factor loading of the K-SMLS compared to the SMLS could be due to the translation process. Although efforts were made to increase validity of the SMLS in the translation process, it was necessary to change the term *advertisement* for the term *promotion*, as cigarette advertisements are illegal in Korea but social media posts promoting smoking or certain new tobacco products are not. However, adolescents would see these social media posts as an advertisement of tobacco products, as they present depictions of smoking. Additionally, the lower factor loading of the K-SMLS could be owed to slight semantic differences among the items (33). Semantic equivalence means “the meaning of each item in each culture is the same after being translated into the language and idiom of each culture” (34), and in a study by Squires et al. (35), it was stated that the criteria for equivalence were not mentioned or were not met, so the semantic equivalence was difficult to determine in translation studies. It is possible that the concepts of smoking media literacy are not fully captured in the items of the K-SMLS because of semantic differences. Furthermore, participants' characteristics could have also made a difference in factor loading. In the study of original SMLS development (17), smokers accounted for 19% of the sample, whereas, in our study, they accounted for only 3.3% of all participants. The smoking rate of participants in this study was lower than the average smoking rate of 6.7% of adolescents in Korea (5), so this could be a biased sample. Bauhoff et al. (36) showed that people who had ever smoked knew more about cigarettes because of advertising compared to never smokers; this could explain the lower factor loading of the K-SMLS, as our sample included only a small proportion of smokers. Therefore, further studies are required to re-validate the scale by including different populations, including current smokers and adolescents with susceptibility to smoking.

Although this study confirmed that the K-SMLS is a valid and reliable instrument to assess SML among Korean adolescents, its limitations should be mentioned. First, this study used a convenient sample from only five schools in a South Korean city. Due to COVID-19, the survey in this study was conducted online, and although frequent reminders were provided to reduce the non-response phenomenon, the response rate in this study was 72.1%. It should be noted that generalization of the results as representative of all Korean adolescents may be limited due to the possibility of sampling bias and non-response bias. Further large-scaled research that conducts a probability sample design such as stratification may compensate to generalize the results to address both possibilities of bias. And further studies need to include participants from diverse socio-demographic backgrounds, including participants living in rural areas and multicultural adolescents. Second, our scale exhibited a relatively low factor loading compared to the original scale, as detailed above. Therefore, in future studies, instruments should supplement words with symbols to convey their meaning more accurately after an instrument is developed through cognitive interviews with adolescents. Third, the number of data points is not sufficient to be divided into two to perform EFA and CFA, so cross-validation has not been performed. In a future study, it will be more helpful to test the factor model identified in this study for different groups.

## Conclusions

SML is a contributor to adolescent smoking. This study translated the SMLS into Korean and tested the validity and reliability of the Korean version. To our knowledge, this is the first study to validate the SMLS with Korean adolescents. It might be possible to perform comparative studies with other countries based on our findings. Further studies should include diverse populations in order to expand the applicability of the K-SMLS.

## Data Availability Statement

The raw data supporting the conclusions of this article will be made available by the authors, without undue reservation.

## Ethics Statement

The studies involving human participants were reviewed and approved by Institutional Review Board of Yonsei University. Written informed consent to participate in this study was provided by the participants and the participants' guardian/next of kin.

## Author Contributions

SK and HL developed the conceptualization of this research and were responsible for the data analysis. HL supervised the research. SK was responsible for data collection and wrote the initial draft of the manuscript. SK, HL, JL, HH, and JK contributed to the interpretation of the results. All authors contributed to the article and approved the submitted version.

## Conflict of Interest

The authors declare that the research was conducted in the absence of any commercial or financial relationships that could be construed as a potential conflict of interest.

## References

[1] World Health Organization. WHO Global Report on Trends in Prevalence of Tobacco Use. 2000-2025. (2019). Available online at: https://www.who.int/publications/i/item/who-global-report-on-trends-in-prevalence-of-tobacco-use-2000-2025-third-edition (accessed February 13, 2021).

[2] American Cancer Society. The Tobacco Atlas. Issue Youth (n.d.). Available online at: https://tobaccoatlas.org/topic/youth/ (accessed February 2, 2021).

[3] Barrington-Trimis JL Leventhal AM. Adolescents' use of “pod mod” e-cigarettes—urgent concerns. New Eng J Med. (2018) 379:1099–102. 10.1056/NEJMp1805758 30134127 PMC7489756

[4] U.S. Department of Health Human Services. Smoking cessation: A report of the Surgeon General. Atlanta, GA: U.S. Department of Health and Human Services (2020). Available online at: https://www.hhs.gov/sites/default/files/2020-cessation-sgr-full-report.pdf (accessed February 2, 2021).

[5] Korea Disease Control and Prevention Agency. The fifteenth Korea Youth Risk Behavior Web-Based Survey Statistics. (2019). Available online at: https://www.cdc.go.kr/yhs/ (accessed February 2, 2020).

[6] OECD. Health at a Glance 2017: OECD Indicators. Paris: OECD Publishing (2017).

[7] Czoli CD White CM Reid JL OConnor RJ Hammond D. Awareness and interest in IQOS heated tobacco products among youth in Canada, England and the USA. Tob Control. (2020) 29:89–95. 10.1136/tobaccocontrol-2018-054654 30696783 PMC7958490

[8] Kang H Cho SI. Heated tobacco product use among Korean adolescents. Tob Control. (2020) 29:466–8. 10.1136/tobaccocontrol-2019-054949 31164491 PMC7361030

[9] Modesto-Lowe V Alvarado C. E-cigs. Are they cool? Talking to teens about e-cigarettes. Clin Pediatr. (2017) 56:947–52. 10.1177/0009922817705188 28443340

[10] Morgenstern M Poelen EA Scholte R Karlsdottir S Jonsson SH Mathis F. Smoking in movies and adolescent smoking: cross-cultural study in six European countries. Thorax. (2011) 66:875–83. 10.1136/thoraxjnl-2011-200489 21873322 PMC3719161

[11] Scull TM Kupersmidt JB Parker AE Elmore KC Benson JW. Adolescents' media-related cognitions and substance use in the context of parental and peer influences. J Youth Adolesc. (2010) 39:981–98. 10.1007/s10964-009-9455-3 19795197 PMC3678372

[12] Lee MS. The smoking image effects in mass media on adolescents. Treatise Plastic Media. (2018) 21:143–9.

[13] Huang J Kornfield R Emery SL. 100 million views of electronic cigarette YouTube videos and counting: Quantification, content evaluation, and engagement levels of videos. J Med Internet Res. (2016) 18:e67. 10.2196/jmir.4265 26993213 PMC4818373

[14] Huang J Kornfield R Szczypka G Emery SL. A cross-sectional examination of marketing of electronic cigarettes on Twitter. Tob Control. (2014) 23:iii26–30. 10.1136/tobaccocontrol-2014-051551 24935894 PMC4078681

[15] Cortese DK Szczypka G Emery S Wang S Hair E Vallone D. Smoking selfies: using Instagram to explore young women's smoking behaviors. Soc Media Soc. (2018) 4:2056305118790762. 10.1177/2056305118790762

[16] Ministry of Health and Welfare and Korean Health Promotion Institute. More Than Half of Dramas, Movies and Webtoons Feature Tobacco and Smoking Scenes. (2019). Available online at: https://www.khealth.or.kr/board/view?pageNum=2&rowCnt=10&no1=433&linkId=999240&menuId=MENU00907&schType=0&schText=&boardStyle=&categoryId=&continent=&country= (accessed February 2, 2021).

[17] Primack BA Gold MA Switzer GE Hobbs R Land SR Fine MJ. Development and validation of a smoking media literacy scale for adolescents. Arch Pediatr Adolesc Med. (2006) 160:369–74. 10.1001/archpedi.160.4.369 16585481 PMC3001232

[18] Vahedi Z Sibalis A Sutherland JE. Are media literacy interventions effective at changing attitudes and intentions towards risky health behaviors in adolescents? A meta-analytic review. J Adolesc. (2018) 67:140–52. 10.1016/j.adolescence.2018.06.007 29957493

[19] Primack BA Douglas EL Land SR Miller E Fine MJ. Comparison of media literacy and usual education to prevent tobacco use: a cluster-randomized trial. J Sch Health. (2014) 84:106–15. 10.1111/josh.12130 25099425 PMC4126196

[20] Ministry of Health and Welfare. Indirect Promotional Activities Such as Providing Discount Vouchers for Electronic Cigarette Devices Are Prohibited. (2020). Available online at: http://www.mohw.go.kr/react/al/sal0301vw.jsp?PAR_MENU_ID=04andMENU_ID=0403andCONT_SEQ=352444andpage=1 (accessed February 2, 2020).

[21] Fielding-Singh P Epperson AE Prochaska JJ. Tobacco product promotions remain ubiquitous and are associated with use and susceptibility to use among adolescents. Nicotine Tob Res. (2021) 23:397–401. 10.1093/ntr/ntaa136 32722775 PMC8269770

[22] World Health Organization. Banning Tobacco Advertising, Promotion and Sponsorship: What You Need to Know. Geneva, Switzerland: WHO Press (2013). Available online at: https://apps.who.int/iris/bitstream/handle/10665/83779/WHO_NMH_PND_13.1_eng.pdf;jsessionid=07D718BC0E75E33015311910C62C2521?sequence=1 (accessed February 2, 2021).

[23] Page RM Piko BF Balazs MA Struk T. Media literacy and cigarette smoking in Hungarian adolescents. Health Educ J. (2011) 70:446–57. 10.1177/0017896910379692 21342356

[24] Page RM Huong NT Chi HK Tien TQ. Smoking media literacy in Vietnamese adolescents. J Sch Health. (2011) 81:34–41. 10.1111/j.1746-1561.2010.00555.x 21158864

[25] World Health Organization. Process of Translation and Adaptation of Instruments. Geneva, Switzerland: WHO Press (n.d.). Available online at: https://www.who.int/substance_abuse/research_tools/translation/en/ (accessed February 2, 2021).

[26] Enforcement Decree of the Tobacco Business Act. Article 10 (Prohibition of Offering Money or Goods, etc. for Promotion of Tobacco Sales): Korea. (2008). Available online at: https://elaw.klri.re.kr/kor_service/lawView.do?hseq=49028&lang=ENG (accessed March 30, 2021).

[27] Polit DF Beck CT Owen SV. Is the CVI an acceptable indicator of content validity? Appraisal and recommendations. Res Nurs Health. (2007) 30:459–67. 10.1002/nur.20199 17654487

[28] Tinsley HE Tinsley DJ. Uses of factor analysis in counseling psychology research. J Couns Psychol. (1987) 34:414–24. 10.1037/0022-0167.34.4.414

[29] Hu LT Bentler PM. Cutoff criteria for fit indexes in covariance structure analysis: conventional criteria versus new alternatives. Struct Equ Model. (1999) 6:1–55. 10.1080/10705519909540118

[30] Kline RB. Principles and Practice of Structural Equation Modeling. 3rd ed. New York, NY: Guilford (2011).

[31] Fornell C Larcker DF. Evaluating structural equation models with unobservable variables and measurement error. J Mark Res. (1981) 18:39–50. 10.1177/002224378101800104

[32] Tabachnick BG Fidell LS. Using Multivariate Statistics. New York, NY: Harper Collins College Publishers (1996)

[33] Krippendorff KH. Content Analysis: An Introduction to Its Methodology. 3rd ed. Thousand Oaks: Sage (2013).

[34] Flaherty JA Gaviria FM Pathak D Mitchell T Wintrob R Richman JA. Developing instruments for cross-cultural psychiatric research. J Nerv Ment Dis. (1988) 176:257–63. 10.1097/00005053-198805000-00001 3367140

[35] Squires A Finlayson C Gerchow L Cimiotti JP Matthews A Schwendimann R. Methodological considerations when translating “burnout”. Burn Res. (2014) 1:59–68. 10.1016/j.burn.2014.07.001 25343131 PMC4203660

[36] Bauhoff S Montero A Scharf D. Perceptions of e-cigarettes: a comparison of adult smokers and non-smokers in a Mechanical Turk sample. Am J Drug Alcohol Abuse. (2017) 43:311–23. 10.1080/00952990.2016.1207654 27712126

