# Corrigendum: Psychometric Properties of the Korean Version of the Smoking Media Literacy Scale for Adolescents

**DOI:** 10.3389/fpubh.2021.731778

**Published:** 2022-01-12

**Authors:** Sookyung Kim, Hyeonkyeong Lee, Jung Jae Lee, Hye Chong Hong, Seungjoo Lim, Junghee Kim

**Affiliations:** ^1^College of Nursing, Yonsei University, Seoul, South Korea; ^2^Mo-Im Kim Nursing Research Institute, College of Nursing, Yonsei University, Seoul, South Korea; ^3^Li Ka Shing (LKS) Faculty of Medicine, School of Nursing, The University of Hong Kong, Hong Kong, China; ^4^Department of Nursing, Chung-Ang University, Seoul, South Korea; ^5^Department of Nursing, Research Institute for Basic Science, Hoseo University, Asan, South Korea; ^6^Department of Nursing, Yonsei University Wonju College of Medicine, Wonju, South Korea

**Keywords:** smoking, media literacy, adolescent, validation, adaptation

In the published article, there was a mistake in the Abstract and Results sections. Instead of Cronbach's alpha = 0.79 in the Abstract, and the Results section, it should be 0.78 to meet the same as Cronbach's alpha in Table 2 and the Discussion section.

The authors apologize for this error and state that this does not change the scientific conclusions of the article in any way. The original article has been updated.

## Publisher's Note

All claims expressed in this article are solely those of the authors and do not necessarily represent those of their affiliated organizations, or those of the publisher, the editors and the reviewers. Any product that may be evaluated in this article, or claim that may be made by its manufacturer, is not guaranteed or endorsed by the publisher.

